# Supplementary material for: Graphene Quantum Dots from Carbonized Coffee Bean Wastes for Biomedical Applications
Source: Nanomaterials (Basel). 2021 May 28;11(6):1423. doi: 10.3390/nano11061423 (PMC8228242; doi:10.3390/nano11061423)
Supplement: Supplementary file 1 [file nanomaterials-11-01423-s001.zip › nanomaterials-1193612-supplementary.pdf]

Supplementary Materials

Graphene Quantum Dots from Carbonized Coffee Bean Wastes for Biomedical Applications

Dong Jin Kim [1,2,3,4,†], Je Min Yoo [3,5,†], Yeonjoon Suh [2], Donghoon Kim [5,6], Insung Kang [7], Joonhee Moon [8], Mina Park [3], Juhee Kim [3], Kyung-Sun Kang [7], and Byung Hee Hong [2,3,*]

1 Program in Nano Science and Technology, Graduate school of Convergence Science and Technology, Seoul National University, Seoul 08826, Korea; dj.kim@snu.ac.kr
2 Graphene Research Center, Advanced Institute of Convergence Technology, Suwon 16229, Korea; yjsuh123@seas.upenn.edu
3 Department of Chemistry, Seoul National University, Seoul 08826, Korea; jyoo3487@bio-graphene.com (J.M.Y.); mn110992@snu.ac.kr (M.P.); juliejjmom@snu.ac.kr (J.K.)
4 Graphene Square Inc., Suwon 16229, Korea
5 BIOGRAPHENE, Los Angeles, CA 90013, USA; dkim@dau.ac.kr
6 Department of Pharmacology, Peripheral Neuropathy Research Center (PNRC), Dong-A University College of Medicine, Busan 49201, Korea
7 Adult Stem Cell Research Center and Research Institute for Veterinary Science, College of Veterinary Medicine, Seoul National University, Seoul 08826, Korea; 95teza@naver.com (I.K.); kangpub@snu.ac.kr (K.-S.K.)
8 Division of Analytical Science Research, Korea Basic Science Institute (KBSI) Daejeon 34133, Korea; junnymoon@kbsi.re.kr
† These authors contributed equally to this work.
* Correspondence: byunghee@snu.ac.kr; Tel.: +82-2-882-6569

AFM image of C-GQDs

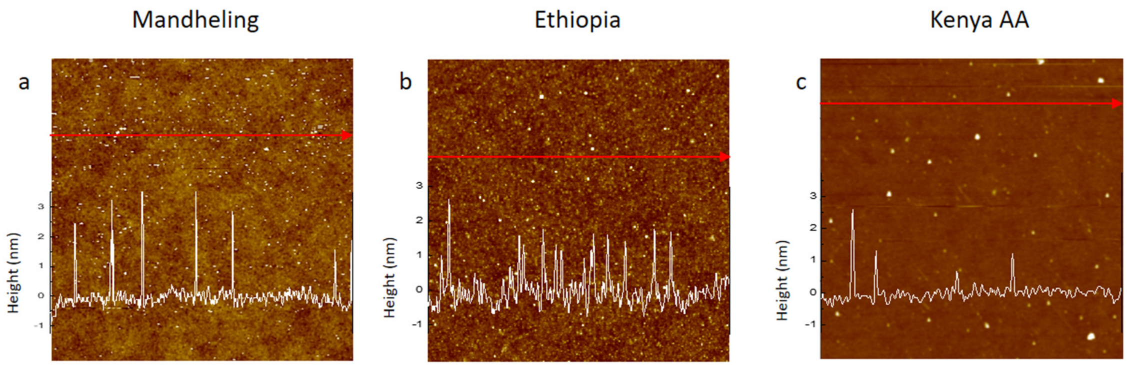

Figure 1. (**a–c**) AFM images of the coffee bean GQDs. Height profiles along the red traversing lines are shown in white graphs.

PL spectra of GQDs.

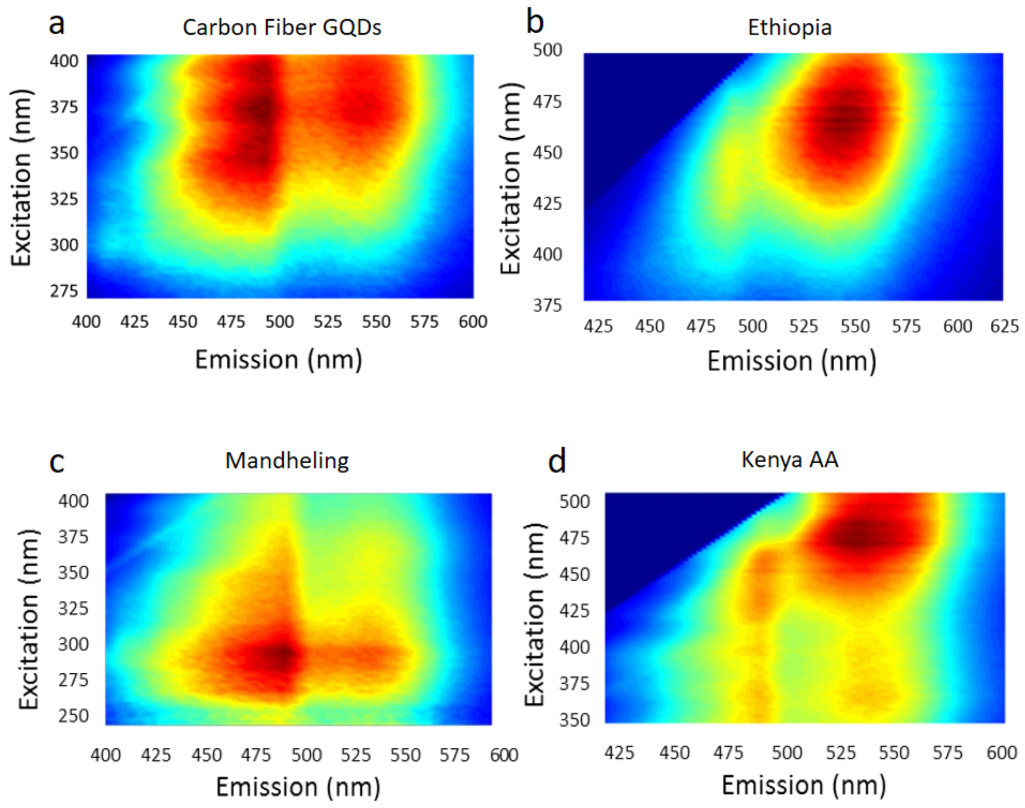

Figure S2. Optical characterization of coffee bean-derived GQDs. (**a**-**d**) PL spectra of the carbon fiber-derived GQ Ds and the coffee bean-derived GQDs.

Optical characterization of coffee bean precursors.

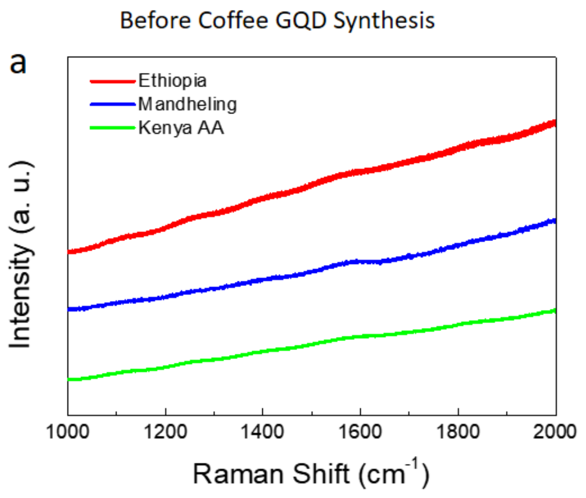

Figure S3. Raman spectra of coffee beans before C-GQD synthesis
